# Optimized anti-reflection core-shell microspheres for enhanced optical trapping by structured light beams

**DOI:** 10.1038/s41598-021-84665-0

**Published:** 2021-03-02

**Authors:** Vahid Shahabadi, Ebrahim Madadi, Daryoush Abdollahpour

**Affiliations:** 1 Department of Physics, Institute for Advanced Studies in Basic Sciences (IASBS), 45137-66731 Zanjan, Iran; 2 Department of Engineering Sciences and Physics, Buein Zahra Technical University, Buein Zahra, 3451745346 Qazvin, Iran; 3 Optics Research Center, Institute for Advanced Studies in Basic Sciences (IASBS), 45137-66731 Zanjan, Iran

**Keywords:** Optical physics, Optical techniques, Optical physics

## Abstract

In this paper, we study the optical trapping of anti-reflection core-shell microspheres by regular Gaussian beam and several structured beams including radially polarized Gaussian, petal, and hard-aperture-truncated circular Airy beams. We show that using an appropriate anti-reflection core-shell microsphere for the optical trapping by several structured light beams can dramatically enhance the strength of the trap compared to the trapping by the common Gaussian beam. The optimal core-shell thickness ratio that minimizes the scattering force is obtained for polystyrene-silica and anatase-amorphous titania microspheres, such that the core-shells act as anti-reflection coated microspheres. We show that the trapping strength of the anti-reflection coated microparticles trapped by the common Gaussian beam is enhanced up to 2-fold compared to that of trapped uncoated microparticles, while the trapping of anti-reflection coated microparticles, by the radially polarized beam, is strengthened up to 4-fold in comparison to that of the trapped uncoated microparticles by the Gaussian beam. Our results indicate that for anatase-amorphous titania microparticles highest trap strength is obtained by radially polarized beam, while for the polystyrene-silica microparticles, the strongest trapping is achieved by the petal beam.

## Introduction

Optical tweezers (OTs)^1–3^ are proven as an indispensable tool for applications in various areas of science ranging from physics to biology such as microscopy^4,5^, investigations of biological cells^6,7^, nanoscale rotary motors^8,9^, and in drug delivery^10,11^. Common OTs is a single Gaussian laser beam that is highly focused at the diffraction-limited spot size by utilizing a high numerical aperture (NA) objective lens. A steep potential well of light intensity at the focus pushes the micro-, nano-particles towards the focal spot, and leads to the formation of a stable optical trap. This radiation pressure exerted on a particle is the consequence of the momentum-transfer of light to the particle^12–15^. The force experienced by particles is Hookian force ($F_i = -k_i\Delta x_i,\ i=x,y,z$) with constant, *k*, representing the trap stiffness^16^. A greater stiffness is an indication of a stronger trap. In general, the net optical force is composed of two different parts: a gradient force, that arises from the intensity gradient of the trapping beam, which drags the particle towards the high-intensity region, and a scattering force that pushes the particle along the propagation direction of the beam. Often, achieving relatively high trap strengths in transverse planes (i.e. perpendicular to the direction of the propagation of the trapping beam) is easier than obtaining adequately strong axial optical traps, and therefore the latter has been a challenging issue in the optical trapping investigations^17,18^.

Optical trap stiffness can be enhanced by either using specific beam shapes or appropriate beads with engineered optical properties, in such a manner to boost the gradient force or to suppress the scattering force. On the one hand, the use of shaped laser beams enhances the trapping forces via minimizing the light scattering^19–23^. Examples include using circular Airy beam for enhanced trapping efficiency of Rayleigh particles^24^, optical trapping with Laguerre–Gaussian modes with improved axial optical forces exerted on the dielectric particles^25^, optical trapping with cylindrical vector beams^21^, creation of enhanced trapping forces with complex-valued Elegant Hermitte- and Laguerre-Gaussian laser beams compared to standard Gaussian beams^26^, and effective multiple optical trapping with petal beams^27^ . On the other hand, the optical and physical properties of the intended particle play an effective role in optical trap enhancement^28–30^. For instance, using hollow gold and silver nanoparticles as handles in the OTs experience stronger optical forces^31^, the effect of resonant mode interference on trapping of gold core-silicon shell nanoparticles have been investigated^32^, optical forces exerted on graphene-coated dielectric particle under the illumination of an arbitrary optical beam have been investigated, theoretically^33^, and plasmonic effects of gold-coated black silicon have shown enhancement of the efficiency of OTs^34^.

Dielectric microspheres have intriguing applications in sensing^35^, viscosity measurements^36^, thermo optical switches^37^, and biocompatible drug delivery^38^. Coated microspheres are shown to result in stronger traps^39,40^, and therefore they have been used as handles in OTs^40^. A core-shell microbead may gain anti-reflection property, with proper choices of materials, and a suitable core-shell diameter ratio, such that the scattering field from the particle is dramatically decreased, and hence a rather strong optical trap is formed^41^. So far, anti-reflection coated polystyrene (PS)-silica and titania (anatase and amorphous) core-shells have been utilized in optical trapping by linearly polarized Gaussian beam, that yielded a few nano-newton optical trap forces^42–44^.

In this paper, we bring together both structured beams and anti-reflection coated microspheres to be used in OTs. We calculate optical trapping forces exerted on core-shell microspheres of PS-silica and anatase-amorphous titania, under illumination by several structured beams such as radially polarized beam (RPB)^45,46^, petal beam (PB)^27,47^, and hard-aperture-truncated circular Airy beam (CAB)^48–50^, and compare them with those of a Gaussian beam commonly used in OTs. Titania core-shell microspheres in two phases (anatase core and amorphous shell), are great candidates to be used in OTs to achieve nano-Newton forces with trap stiffness greater than $1~\mathrm { pN\,nm^{-1}}$. Following^43^, we use a $1~{\upmu \hbox {m}}$ radius titania core-shell microsphere with a variable anatase core. Additionally, PS-silica core-shell microsphere with the same total radius, and variable core-shell radius ratios is considered. We use Debye diffraction theory^51^ along with the generalized Lorenz–Mie theory (GLMT), and Maxwell stress tensor^52^ to numerically calculate the optical forces on the anti-reflection-coated microspheres.

## Methods

In this section, we briefly discuss the properties of the anti-reflection-coated microspheres. Additionally, the mathematical formulations of the intended structured beams are introduced. Finally, we present the physical and mathematical basis for the calculations of the optical forces under the formalism of GLMT.

**Figure 1:**
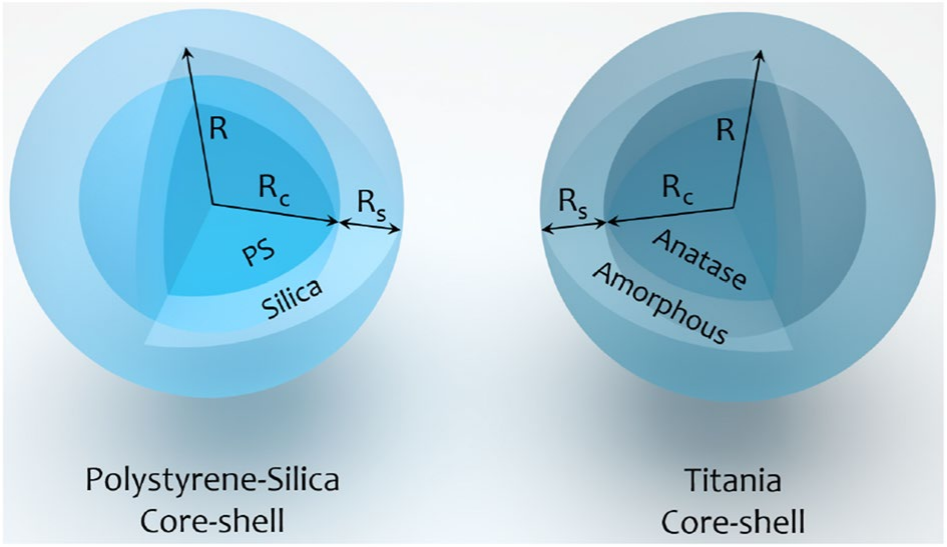
Schematic structures of a PS-silica and a titania core-shell microspheres. $R_s$, $R_c$, and *R* are the shell thickness, core radius, and the total radius of the microsphere ($R = R_c + R_s$). This figure is generated using Sketchup 2020.

### Anti-reflection-coated microspheres

Structure of anti-reflection core-shell microspheres of PS-silica and titania are illustrated in Fig. 1. The refractive indices of the core, and the shell, $n_c,\ n_s$, respectively, are given in Table 1, while the surrounding medium is considered to be water with a refractive index of $n_{m}=1.33$. Inner and outer radii of the core-shell microsphere are $R_c$ and *R*, respectively. we can use the refractive index of the anti-reflection material for a single anti-reflection layer of a planar surface for a shell in spherical geometry^43,44^ which is:

1 $$\begin{aligned} n_{s} \approx \sqrt{n_{c} n_{m}}. \end{aligned}$$

while by this choice, the optimum thickness of the anti-reflection coating of a planer surface, $R_s = \uplambda /4n_s$ is not reliable in the core-shell microsphere case and it should be calculated through Mie scattering theory^43^.

**Table 1:** Refractive indices of core-shell microspheres^42,43^.

Core-shell material	$n_c$	$n_s$
PS-silica	1.57	1.45
Anatase-amorphous titania	2.3	1.8

### Structured light beams

#### Radially polarized beam

Radially polarized beam (RPB) is categorized as a cylindrical vector beam in which the polarization direction is radially symmetric in a lateral plane of a cylindrical coordinate system. This symmetrical polarization distribution results in a polarization singularity on the beam axis which leads to a dark spot on the axis. RPB can be expressed as a superposition of two orthogonal lowest order Hermite–Gaussian modes with orthogonal polarizations ($z = 0$)^45^

2 $$\begin{aligned} {\mathbf {E}}_{\mathrm {RPB}}(x,y) =E_0 \left( {\mathrm {HG}}_{10}(x,y){\hat{\mathbf{x}}} + {\mathrm {HG}}_{01}(x,y){\hat {\mathbf{y}}} \right) , \end{aligned}$$

where $\mathrm {HG}_{mn}(\cdot )$ are the Hermite–Gaussian polynomials, and *x*, *y* are the Cartesian coordinates in the transverse plane.

#### Petal beam

Coherent superposition of two collinearly propagating Laguerre–Gaussian beams with opposite sign azimuthal topological charge (*l*) and zero radial topological charge ($p = 0$), generates a new class of structured light beams known as petal beams (PB) (also known as cogwheel beams^47^) because of the petal-like field distribution in the lateral plane ($z = 0$)^53^

3 $$\begin{aligned} {\mathbf {E}}_{\mathrm {PB}}(\rho ,\varphi ) = E_0\left( \mathrm {LG}_{p=0}^l(\rho , \varphi ) +\mathrm {LG}_{p=0}^{-l}(\rho , \varphi )\right) \varvec{{\hat{e}}}, \end{aligned}$$

where $\mathrm {LG}_{p}^l(\cdot )$ are the Laguerre-Gaussian polynomials, $\rho$ and $\varphi$ are the radial and azimuthal coordinates in the transverse plane, and $\varvec{{\hat{e}}}$ is the polarization state.

#### Circular airy beam

Circular Airy beam (CAB) is categorized in a new family of beams known as abruptly autofocusing beams, possessing a special capability of autofocusing without an external element. The electric field envelope of CAB at an initial propagation distance ($z=0$) is given as^48,50^

4 $$\begin{aligned} {\mathbf {E}}_{\mathrm {CAB}}(\rho ) =E_0 \mathrm {Ai}\left( \frac{\rho _0 - \rho }{w}\right) \mathrm {exp}{\left( a\frac{\rho _0 - \rho }{w}\right) }\varvec{{\hat{e}}}, \end{aligned}$$

where $\mathrm {Ai}(\cdot )$ denotes the Airy function. $\rho$ is a radial coordinate in the transverse plane, $\rho _{0}$ is the radius of the main ring of the beam, *a* is a decay factor, *w* is a scaling factor that determines the width of the main ring, and $\varvec{{\hat{e}}}$ is the polarization state.

**Figure 2:**
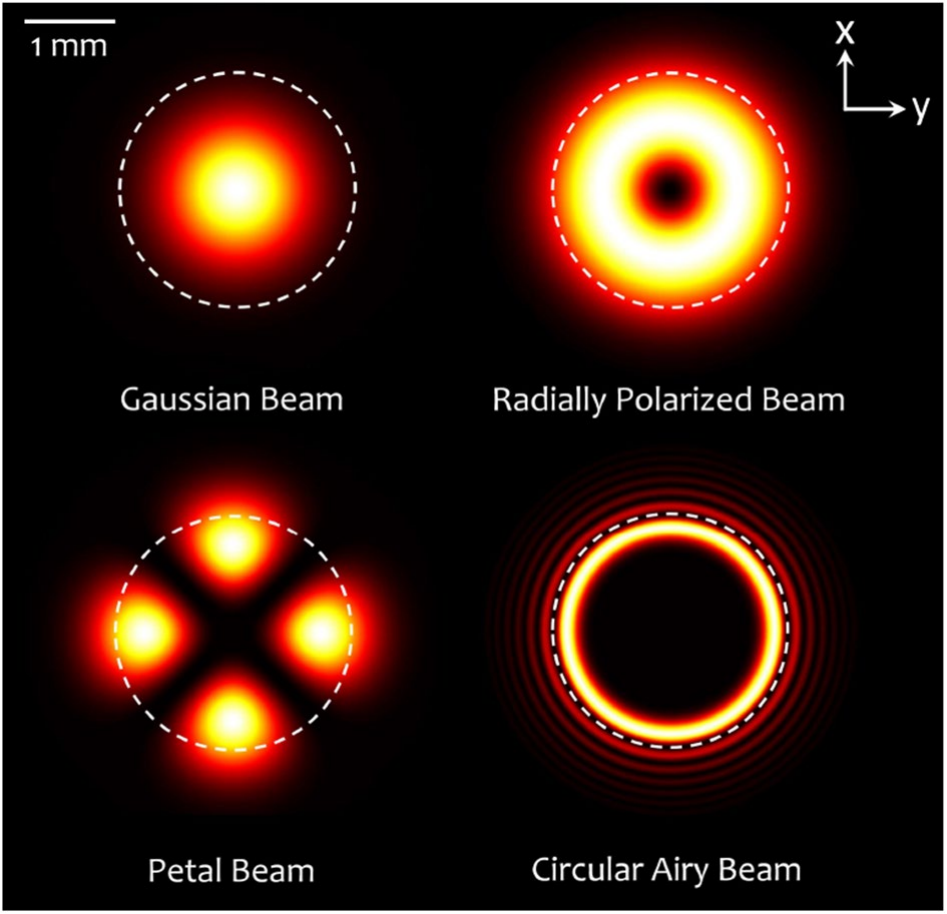
Intensity profiles of the intended structured light beams at $z = 0$ plane for filling ratio ($w_0/R_o$) equal to 0.8 ($w_0 = 1~\mathrm {mm}$ and $R_o = 1.25~\mathrm {mm}$). Dashed circles show the radius of the back aperture of the objective lens $R_o$. The Gaussian, petal, and circular Airy beams are right-handed circularly polarized. This figure is generated using Octavev.6.1.0.

Figure 2 illustrates the intensity distributions of a Gaussian Beam (GB), RPB, PB ($l = 2$), and CAB. GB, PB, and RPB have identical initial waist of $w_0 = 1~\mathrm {mm}$. Moreover, CAB parameters are set as $\rho _0 = 1~\mathrm {mm}$, $w = 0.095~\mathrm {mm}$, and $a = 0.15$, since an equivalent envelope Gaussian beam to CAB is defined to have a waist equal to the radius of the main ring of the CAB (i.e. $\rho _0 = w_0$)^54^. Furthermore, GB, PB and CAB are assumed to be circularly polarized, and all beams are set to convey an identical power.

**Figure 3:**
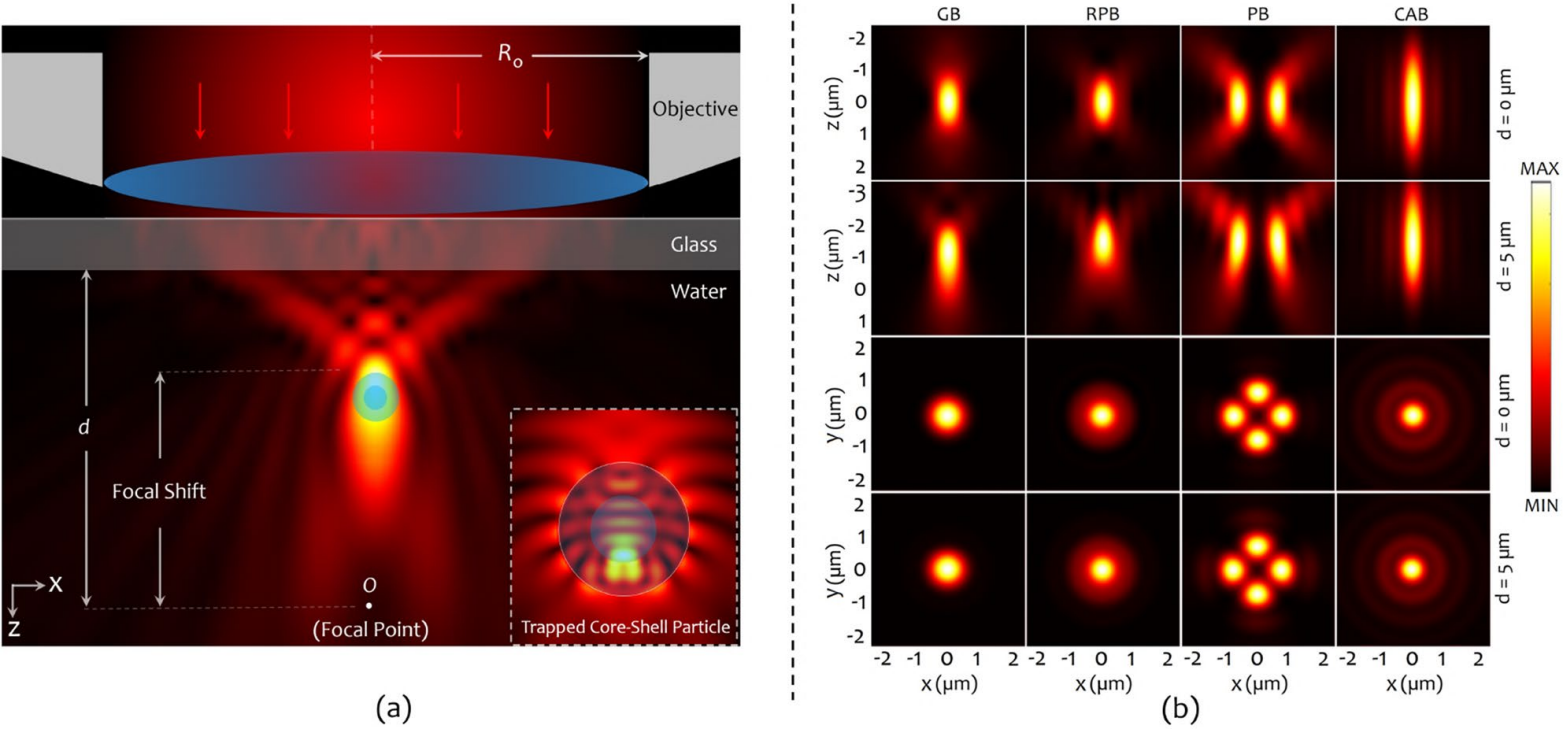
(**a**) Schematic of the focusing process of an optical beam by an objective lens, and the intensity distribution around a particle. The induced spherical aberration, due to glass-water refractive index mismatch shifts the focus towards the interface. (**b**) Calculated intensities of the focused beams in the axial and lateral directions in the absence of the spherical aberration, $d = 0$, (first and third rows), and in the presence of the spherical aberration, $d = 5~{\upmu \hbox {m}}$, (second and forth rows). Columns 1 to 4, from left to right, show the intensity distributions of the GB, RPB, PB, and CAB, respectively. This figure is generated using Octavev.6.1.0 and Apache OpenOffice 4.1.8.

### Beam focusing

In OTs setups, an optical beam is tightly focused by a high numerical aperture (NA) oil-immersion objective lens. The beam passes through a lam (coverglass) and enters the trapping medium (water), as shown in Fig. 3a. With the origin of a Cartesian coordinate system located at the focal point of the objective lens, the focal field distribution can be calculated by using the vectorial Debye diffraction theory, as^51^:

5 $$\begin{aligned} \mathbf{E }_f(\mathbf{r })= \frac{ikfe^{-ikf}}{2\pi }\int _{0}^{\theta _{max}}\sin \theta \int _{0}^{2\pi } \mathbf{E }_\text {ff,t}(\theta ,\varphi )e^{i\mathbf{k }.\mathbf{r }}e^{i\xi }d\varphi \,d\theta , \end{aligned}$$

where *k*, *f*, $\theta _{max}$ are the wavenumber, focal length of the objective, and maximum converging angle ($60^{\circ }$, equivalent to NA = 1.3), respectively. According to^55^, the optimal filling ratio, defined as the ratio of the beam waist to back-aperture radius of the focusing objective $R_{o}$, (i.e. $w_0/R_o$) is taken to be 0.8. The back-aperture radius of the objective lens is depicted by the dashed circles in Fig. 2. It is important to note that with such an arrangement, the back-aperture of the objective truncates the CAB in a manner that only its first ring is transmitted through the lens. $\mathbf{E }_\text {ff,t}$ is the far-field distribution of the optical beams, given by Eqs. (2)–(4), transformed from the cylindrical coordinate system before the lens to a spherical coordinate system with the origin at the focus:

6 $$\begin{aligned} {\mathbf {E}}_\text {ff,t}(\theta ,\varphi ) = \left( t_s(\theta ) \left[ {\mathbf {E}}_\text {i}(\rho ,\varphi ) \ .\ \varvec{{\hat{\varphi }}}\right] \varvec{{\hat{\varphi }}} + \ t_p(\theta ) \left[ {\mathbf {E}}_\text {i}(\rho ,\varphi ) \ .\ \varvec{{\hat{\rho }}}\right] \varvec{{\hat{\theta }}}\right) \sqrt{\frac{\cos \theta }{n_1}}, \end{aligned}$$

where $t_s(\theta )$ and $t_p(\theta )$ are the Fresnel’s transmission coefficients of the objective for perpendicular and parallel polarization states, respectively. The refractive index mismatch through the glass-water interface induces a spherical aberration $\xi = k_0d(n_1\cos \theta _1 - n_2\cos \theta _2)$, in the system^56^, where $k_0 = 2\pi /\uplambda$ is the vacuum wave number ($\uplambda = 1064~\mathrm {nm}$), *d* is the distance between the glass and the focal point (*O*); $n_1 = 1.52$ and $n_2 = 1.33$, are the refractive indices of the glass and water, and $\theta _1$ and, $\theta _2$ are the convergence angles in the glass and water, respectively, which are related through the Snell’s law of refraction.

Figure 3b shows the intensity distributions of the focused beams in the axial and lateral directions, in the absence of the spherical aberration, i.e. $d = 0$, (first and third rows), and in the presence of the spherical aberration, i.e. $d = 5~{\upmu \hbox {m}}$ (second and fourth rows). As can be seen, the spherical aberration shifts and the focus towards the interface (by about 1–$1.5~{\upmu \hbox {m}}$), and also disturbs the intensity distribution. The axial FWHMs of the focused beams are approximately $1.3\uplambda$, $\uplambda$, $1.2\uplambda$ and 1.9*rlambda* for GB (first column), RPB (second column), PB (third column), and CAB (fourth column), respectively, in the presence of the aberration. In the lateral direction, on the other hand, the intensity distributions are less affected by the spherical aberration. The lateral FWHMs are approximately $0.5\uplambda$, $0.46\uplambda$, $0.41\uplambda$ and $0.4\uplambda$ for GB, RPB, PB, and CAB, respectively, in the presence of the aberration.

### Optical force calculation

To calculate the net optical force exerted on a particle, it is necessary to calculate scattered fields from the particle. The GLMT is used to calculate the scattered fields from the trapped particle. In this method, the incoming and scattered fields are expanded in terms of vector spherical harmonics^57,58^:

7 $$\begin{aligned} {\mathbf {E}}_i= & {} \sum _{pnm}{\mathcal {W}}_{nm}^{(p)}{\mathbf {J}}_{nm}^{(p)}, \quad \quad {\mathbf {E}}_s = \sum _{pnm}{\mathcal {A}}_{nm}^{(p)}{\mathbf {H}}_{nm}^{(p)} , \end{aligned}$$

where ${\mathcal {W}}_{nm}^{(p)}$ and ${\mathcal {A}}_{nm}^{(p)}$ are the expansion coefficients of the incoming and scattered fields, respectively. ${\mathbf {J}}_{nm}^{(p)}$ and ${\mathbf {H}}_{nm}^{(p)}$ are multipoles for incoming and scattered fields, respectively which are expanded on the basis of vector spherical harmonics. Here, $p=1,2$, represent the $\text {TM}$ and $\text {TE}$ fields, respectively. By multipole expansion of focused field in Eq. (5), the expansion coefficients of the incoming focused beam can be calculated. Then, the expansion coefficients of the scattered field can be obtained by applying the boundary conditions on the surface of the particle:

8 $$\begin{aligned} {\mathcal {A}}_{n'm'}^{(p')} = \sum _{p=1,2}\sum _{n=0}^{n_{max}}\sum _{m=-n}^{n} {\mathcal {T}}_{n'm'\ nm}^{(p' p)}{\mathcal {W}}_{nm}^{(p)}, \end{aligned}$$

where ${\mathcal {T}}_{n'm'\ nm}^{(p'p)}$ is the transfer matrix of the particle, which reduces to the Mie coefficients for a single spherical particle^58^:

9 $$\begin{aligned} {\mathcal {A}}_{nm}^{(1)}= & {} b_{n} {\mathcal {W}}_{nm}^{(1)},\quad \qquad {\mathcal {A}}_{nm}^{(2)} = a_{n} {\mathcal {W}}_{nm}^{(2)}. \end{aligned}$$

Here, Mie coefficients of core-shell microspheres can be expressed as^59^:

10 $$\begin{aligned} a_n= & {} \frac{\Psi _n(y)[\Psi _{n}^{'}(\eta _2y) - A_n\chi _{n}^{'}(\eta _2y)] - \eta _2 \Psi _n^{'}(y)[\Psi _{n}(\eta _2y) - A_n\chi _{n}(\eta _2y)]}{\Phi _n(y)[\Psi _{n}^{'}(\eta _2y) - A_n\chi _{n}^{'}(\eta _2y)] - \eta _2 \Phi _n^{'}(y)[\Psi _{n}(\eta _2y) - A_n\chi _{n}(\eta _2y)]},\nonumber \\ b_n= & {} \frac{\eta _2\Psi _n(y)[\Psi _{n}^{'}(\eta _2y) - B_n\chi _{n}^{'}(\eta _2y)] - \Psi _n^{'}(y)[\Psi _{n}(\eta _2y) - B_n\chi _{n}(\eta _2y)]}{\eta _2\Phi _n(y)[\Psi _{n}^{'}(\eta _2y) - B_n\chi _{n}^{'}(\eta _2y)] - \Phi _n^{'}(y)[\Psi _{n}(\eta _2y) - B_n\chi _{n}(\eta _2y)]}, \end{aligned}$$

with

11 $$\begin{aligned} A_n= & {} \frac{\eta _2\Psi _n(\eta _2x)\Psi _n^{'}(\eta _1x) - \eta _1\Psi _n^{'}(\eta _2x)\Psi _n(\eta _1x)}{\eta _2\chi _n(\eta _2x)\Psi _n^{'}(\eta _1x) - \eta _1\chi _n^{'}(\eta _2x)\Psi _n(\eta _1x)}, \nonumber \\ B_n= & {} \frac{\eta _2\Psi _n(\eta _1x)\Psi _n^{'}(\eta _2x) - \eta _1\Psi _n^{'}(\eta _1x)\Psi _n(\eta _2x)}{\eta _2\chi _n^{'}(\eta _2x)\Psi _n(\eta _1x) - \eta _1\chi _n(\eta _2x)\Psi _n^{'}(\eta _1x)}, \end{aligned}$$

where $\eta _1 = n_c/n_2$ and $\eta _2 = n_s/n_2$. $x = kR_c$ and $y = kR$ are the size parameters (*k* is the wave number in the ambient medium).

12 $$\begin{aligned} \Psi _n(x) = xj_n(x), \quad \chi _n(x) = xy_n(x), \quad \Phi _n(x) = xh_n^{(1)}(x) = \Psi _n(x)+i\chi _n(x), \end{aligned}$$

where $j_n$ and $h_n^{(1)}$ are the Bessel and the first kind of Hankel functions, respectively. $\Psi _n(x)$, $\chi _{n}(x)$ and $\Phi _n(x)$ are the Riccati–Bessel functions.

The optical force exerted on the particle can be calculated by numerical integration over time-averaged Maxwell stress tensor^57^:

13 $$\begin{aligned} \mathbf{F } = \oint _S \left\langle {\mathcal {T}}_{\mathbf{M }}\right\rangle .\hat{\mathbf{n }} \ dS, \end{aligned}$$

where *S* represents a surface encircling the particle, and $\hat{\mathbf{n }}$ is a unit vector normal to *S*, respectively. ${\mathcal {T}}_{\mathbf{M }}$ is the Maxwell stress tensor defined as

14 $$\begin{aligned} {\mathcal {T}}_{\mathbf{M }} = \frac{\varepsilon _0}{2}\left[ n_2^{2}{\mathbf {E}}\otimes \mathbf {E^*} + c^2{\mathbf {B}}\otimes \mathbf {B^*}-\frac{{\mathbf {I}}}{2}(n_2^{2}\left| E \right| ^2 + c^2\left| B \right| ^2) \right] , \end{aligned}$$

where *c* and $\varepsilon _0$ are the speed of light and permittivity of vacuum, respectively. The symbol $\otimes$ denotes dyadic multiplication, and ${\mathbf {I}}$ is the unit dyadic. Additionally, ${\mathbf {E}} = {\mathbf {E}}_i + {\mathbf {E}}_s$ and ${\mathbf {B}} = {\mathbf {B}}_i + {\mathbf {B}}_s$, are the total electric and magnetic fields, respectively, which are interrelated through $\mathbf{B } = (i/\omega ) \nabla \times \mathbf{E }$, with $\omega$ representing the angular frequency of the light.

**Figure 4:**
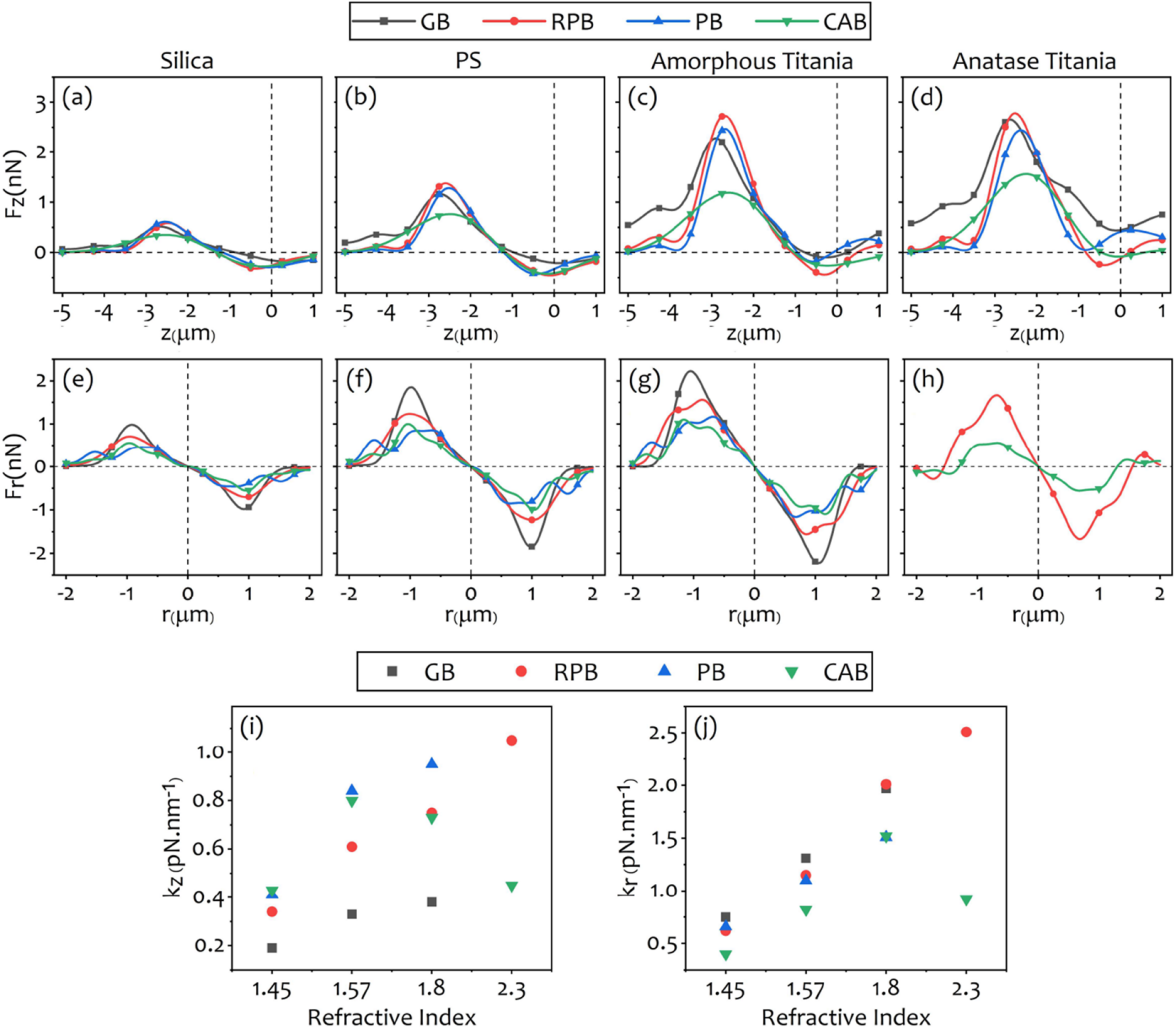
Axial (**a**–**d**) and lateral (**e**–**h**) force diagrams of structured light beams exerted on the uniform microspheres ($R = 1~{\upmu \hbox {m}}$) made up of 4 intended materials and comparison with the Gaussian beam. Axial (**i**) and lateral (**j**) trapping stiffnesses of each particle trapped by the structured beams.

## Results and discussion

Here, we summarize the results of our calculations based on above mentioned theoretical foundations for uncoated uniform and anti-reflection-coated microparticles. In our calculations, the GB, RPB, PB and, CAB are used for optical trapping. The trapping wavelength and the power in the entrance pupil are assumed to be $1064~\mathrm {nm}$ and $1~\mathrm {Watt}$, respectively. Moreover, all calculations are done for a depth of $d = 5~{\upmu \hbox {m}}$ and a filling ratio of 0.8. The total radius of the particles are chosen to be constant and equal to $R = 1~{\upmu \hbox {m}}$.

First, we report the calculation results for uniform microspheres of PS, silica, amorphous-, and anatase-titania with refractive indices outlined in Table 1. Figure 4 shows the axial (Fig. 4a–d) and lateral trapping forces (Fig. 4e–h) exerted on different uniform beads by the structured beams. The calculated trap stiffnesses, along the axial and lateral directions, versus the refractive index of the uniform beads are shown in Fig. 4i,j, respectively. Trap stiffness can be achieved by calculating the slope of a line fitted on the curves of forces versus displacements in the longitudinal and transverse dimensions (i.e. $\mathrm {F_z-z}$, and $\mathrm {F_r-r}$ curves) at the linear region, around the equilibrium position of the trapped particle. Obviously, the beads with higher refractive indices experience greater maximum axial forces for all beams. The maximum axial forces rises from $\sim 0.5~\mathrm {nN}$ for the silica bead with the lowest refractive index to $\sim 2.5~\mathrm {nN}$ for the anatase-titania bead with the highest refractive index. The trap stiffness increases as the refractive index of micro-bead increases for all structured beams except for the CAB whose axial (lateral) trapping stiffness is maximum for the bead with the refractive index equal to 1.57 (1.8), as can be seen in Fig. 4i,j. It is obvious from Fig. 4d that the GB and PB are unable to trap the anatase because of the large refractive index difference of anatase with the ambient while the RPB provides a stable trap with an axial stiffness greater than $1~\mathrm {pN\,nm^{-1}}$, as seen in Fig. 4i, and a lateral stiffness greater than $2.5~\mathrm {pN\,nm^{-1}}$, as is seen in Fig. 4j . Almost for all beads, the RPB yields the largest axial force in comparison with the other beams, while the truncated CAB has the smallest axial force. It is seen that, with an exception of trapping the anatase bead, structured beams provide $\sim 2$- to 2.5-fold stronger traps than that of the GB (see Fig. 4i). In the lateral direction, the GB provides the greatest force (except for the anatase). This is attributed to a greater lateral intensity gradient of the GB compared to that of the structured beams. The figure also shows that the lateral stiffness for the GB is about 4-fold greater than that of the axial direction, while the lateral stiffnesses for the structured beams, in the best case, are about 2-fold greater than the corresponding axial stiffnesses. For instance, the strongest trap is provided by the RPB with a lateral stiffness greater than $2.5~\mathrm {pN\,nm^{-1}}$ which is $\sim 2.5$-fold greater than the corresponding value in the axial direction (see Fig. 4j). Among all beams, only the RPB and the CAB can form a three-dimensional trap for the high refractive index micron-sized uncoated particles in the presence of the spherical aberration. It is also worth noting that the RPB produces the strongest trap due to its steeper intensity gradient^60^.

**Figure 5:**
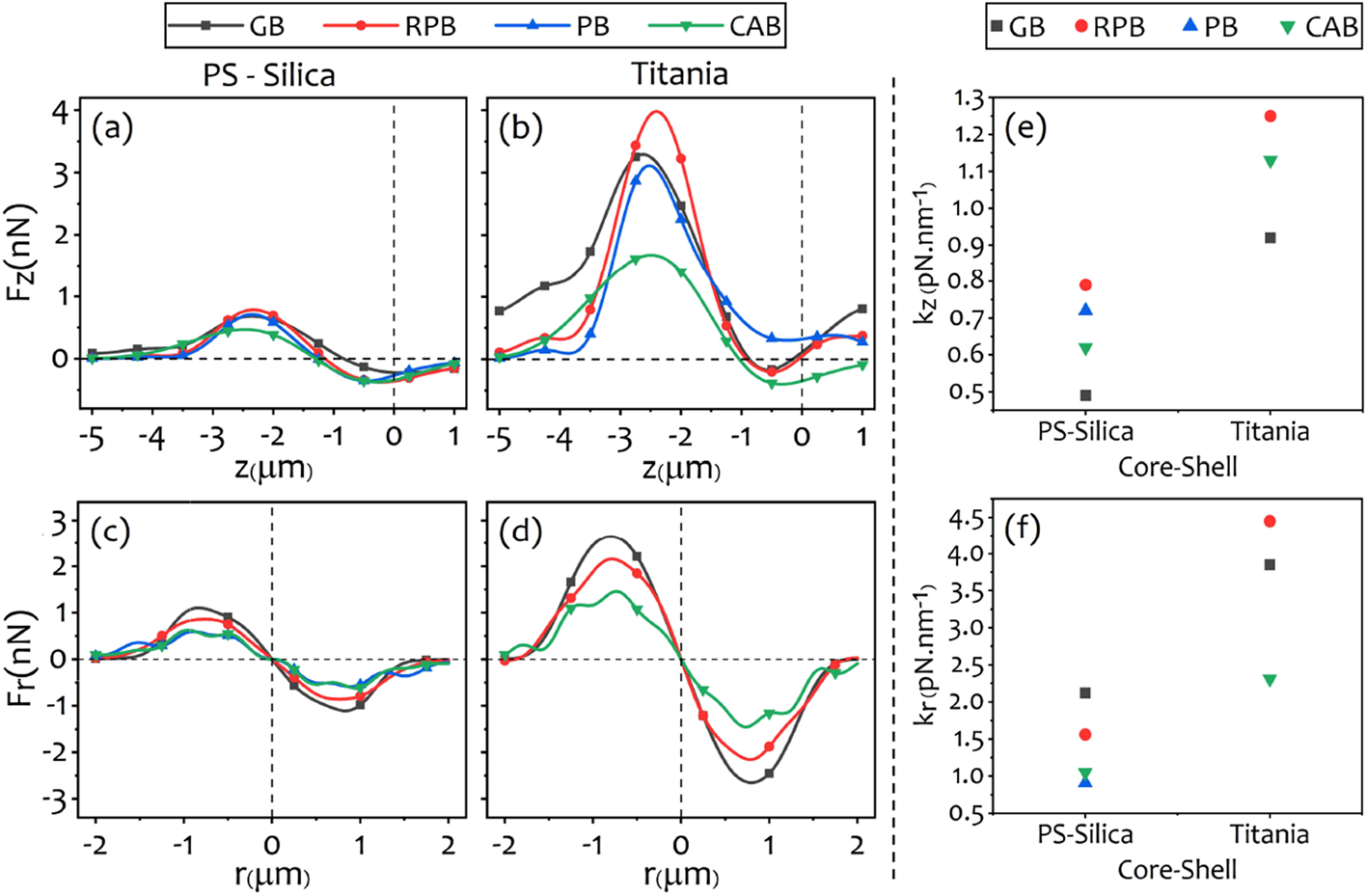
Axial (**a**,**b**) and lateral (**c**,**d**) force diagrams for PS-silica and titania core-shells with the same core and shell sizes ($R_c = R_s = 0.5~{\upmu \hbox {m}}$). Axial and lateral calculated stiffness values are shown in (**e**,**f**), respectively.

Anti-reflection coating on the microspheres reduces the scattering fields that destabilize the optical traps. Figure 5 shows the results of the calculations for optical trapping of PS-silica and anatase-amorphous titania core-shell microspheres by the structured beams. Here, the core-shell microspheres used in our calculations have the same core radius and shell thickness (i.e. $R_c = R_s = 0.5~{\upmu \hbox {m}}$). It is clearly seen that the RPB creates the greatest maximum axial force (Fig. 5a,b) compared to other beams due to its sharp focus in the axial direction. For instance, the maximum axial optical force is about $4.1~\mathrm {nN}$ for titania core-shell, which shows $\sim 46\%$ enhancement in comparison with the maximum axial optical force exerted on a uniform anatase microsphere shown in Fig. 4d. Figure 5e shows that the trap stiffness of the PS-silica microsphere trapped by the GB and RPB is enhanced compared to that of the PS microsphere shown in Fig. 4i. Qualitatively, trap stiffness for the uniform PS microsphere, trapped by the GB and the RPB are about 0.33 and $0.61~\mathrm {pN\,nm^{-1}}$, respectively, while the corresponding values for PS-silica are 0.49 and $0.79~\mathrm {pN\,nm^{-1}}$ that means $\sim 50\%$ and $\sim 30\%$ increase in the trap stiffness, respectively. However, the PB and CAB do not represent enhancement in this case. In the lateral direction, the GB creates the greatest maximum exerted optical force for both PS-silica and titania core-shells (Fig. 5c,d). The lateral trapping stiffness for PS-silica and titania particles are shown in Fig. 5f. For PS-silica particles, a comparison with the corresponding values of the trap stiffnesses plotted in Fig. 4j for PS, reveals an enhancement of $\sim 60\%$, $\sim 36\%$, and $\sim 25\%$ for the GB, RPB, and CAB, respectively.

The most pronounced message of Fig. 5b compared to Fig. 4d is the ability of the GB to trap titania core-shell microsphere. Also, Fig. 5e reveals that the axial stiffness for titania core-shell trapped by the GB is increased by a factor of 2 compared to that of amorphous titania (Fig. 4i). Furthermore, it can be seen that the axial stiffness of the RPB increases from $1.05~\mathrm {pN\,nm^{-1}}$ in Fig. 4i, up to $1.25~\mathrm {pN\,nm^{-1}}$ in Fig. 5e, which is about $\sim 20\%$ enhancement when we use titania core-shell rather than the uniform anatase microsphere. In addition, it shows that the strength of trapping of the titania core-shell with the CAB is enhanced by about $\sim 150\%$ and $\sim 55\%$ compared to the trapping of anatase and amorphous titania microspheres, respectively. On the other hand, lateral stiffness enhances about $\sim 78\%$ for the RPB, and more than $\sim 150\%$ for the CAB in comparison with corresponding lateral stiffness for anatase titania illustrated in Fig. 4j. Figure 5b also shows that here again the PB is unable to trap the titania core-shell microsphere.

Although, Fig. 5 represents a significant enhancement in trapping strength of the core-shell microspheres with the same core radius and shell thickness, compared to trapping of the uniform microspheres, but it might not be an optimized configuration for achieving an efficient anti-reflection property. In order to investigate a configuration that may result in an optimized anti-reflection property of a coated microsphere, that reduces the scattered fields by the particles, we consider the core radius as a variable and define a ratio of the core radius to the total radius of the microsphere as a new parameter $\alpha$ as:

15 $$\begin{aligned} \alpha = R_c/R. \end{aligned}$$

We then repeat the calculations for different values of $\alpha$ to find an optimal value that yields maximum enhancements of the axial and lateral trap stiffnesses for each of the structured beams.

**Figure 6:**
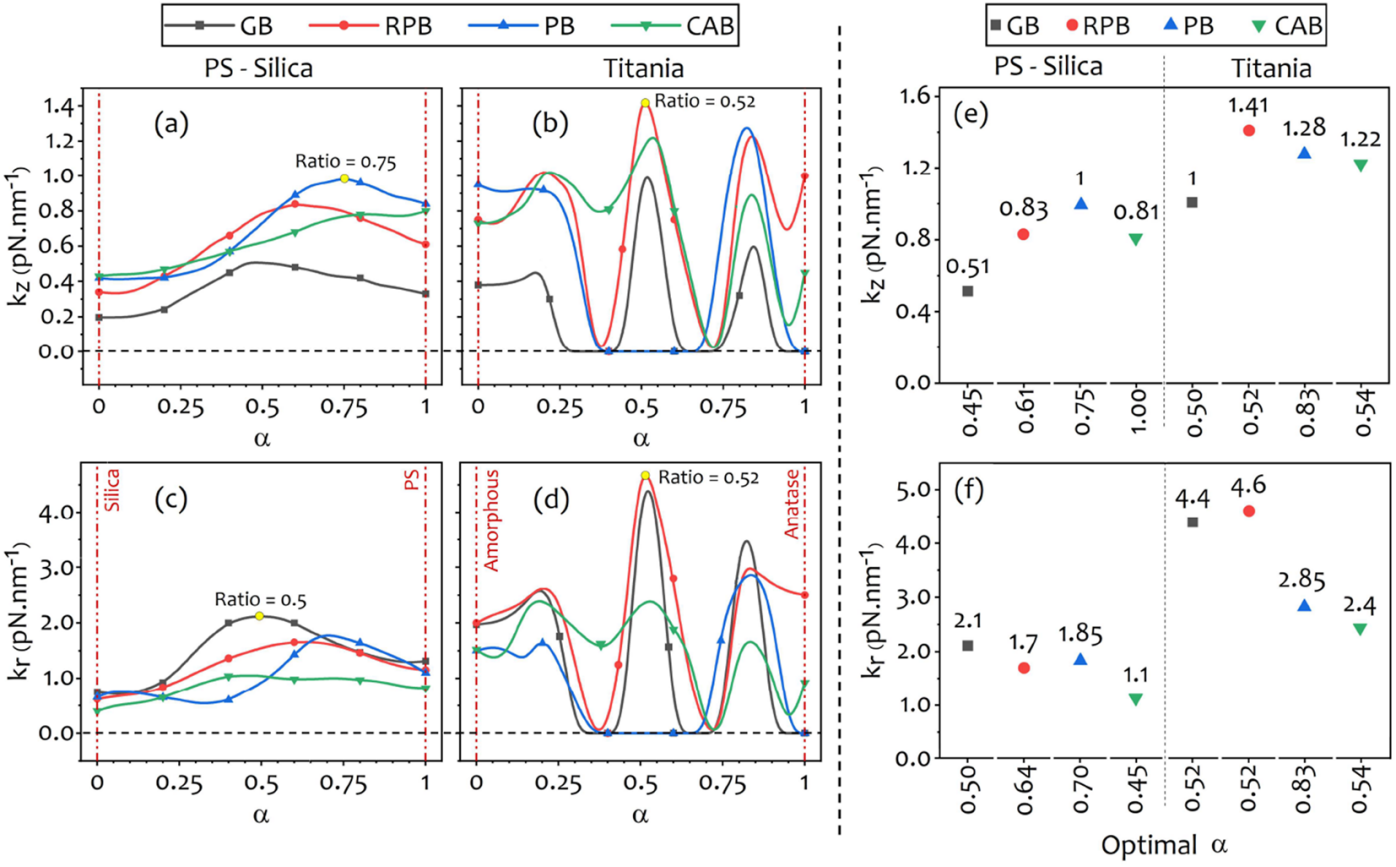
The calculated axial stiffness values vs. $\alpha$ for PS-silica (**a**) and titania core-shells (**b**), and lateral stiffness values vs. $\alpha$ for PS-silica (**c**) and titania core-shells (**d**), and specify corresponding $\alpha$ to the most enhanced case. The axial (**e**) and lateral (**f**) stiffness values vs optimal $\alpha$ for PS-silica and titania core-shells.

Figure 6 illustrates the calculated trap stiffnesses for different values of $\alpha$ in the full range from $\alpha =0$ (i.e. uniform silica or amorphous titania) to $\alpha =1$ (i.e. uniform PS or anatase titania). It is clear that the strongest axial trap for trapping PS-silica microparticle belongs to the PB with $\alpha =0.75$ (it means the radius of PS core equals $0.75~{\upmu \hbox {m}}$). The stiffness value is about $\sim 1~\mathrm {pN\,nm^{-1}}$ that is $\sim 20\%$ higher than the corresponding value for the uniform PS particle trapped by the PB, and 3-fold, 5-fold, and 2-fold greater than those of the PS, silica, and PS-silica with $\alpha =0.5$ microspheres trapped by the GB. Figure 6a shows that the structured light beams provide stronger traps along the axial direction than the GB in all the range of $\alpha$. It can be seen from Fig. 6c,f that the GB yields the strongest lateral trap for trapping the PS-silica microparticle with $\alpha = 0.5$ with a stiffness value equal to $\sim 2.12~\mathrm {pN\,nm^{-1}}$, which is about $\sim 62\%$ and $\sim 180\%$ greater than the corresponding values for trapping the uniform PS and silica microspheres with the GB, respectively.

Looking at Fig. 6b,d for axial and lateral trapping stiffnesses of the titania core-shell, reveals that (1) the behavior of trapping stiffness is not simple and have some maxima and minima, and (2) the maximum values of trapping stiffnesses are approximately independent of the beam type, and the peaks appear at the same values of $\alpha$ for all beam types. This implies that for the titania core-shell particle, certain values of $\alpha$ considerably minimize the scattering fields while for other values the scattering fields are strong enough to destabilize the optical traps, disregarding the beam type. Moreover, the RPB provides the strongest and the most enhanced axial trap strength for $\alpha = 0.52$ with an axial stiffness value $\sim 1.4~\mathrm {pN\,nm^{-1}}$, which is $\sim 40\%$ higher than that of the uniform anatase microsphere trapped by the RPB, $\sim 48\%$ higher than that of uniform amorphous titania trapped by the PB (both shown in Fig. 4i), and $\sim 40\%$ higher than that of the titania core-shell trapped by the GB (Fig. 6e). Our results for trapping the titania core-shell is in good agreement with the results of Jannasch et al.^43^. In order to compare our results, the maximum reported lateral and axial trap stiffnesses in their work are $3.8~\mathrm {pN\,nm^{-1}}$ and $0.9~\mathrm {pN\,nm^{-1}}$, while we obtained $4.6~\mathrm {pN\,nm^{-1}}$ and $1.41~\mathrm {pN\,nm^{-1}}$, respectively, which show more than 20$\%$ and 56$\%$ enhancement for trapping by utilizing the RPB instead of the GB. Furthermore, as Fig. 6f shows, the RPB produces the greatest trapping stiffness for trapping the titania core-shell with $\alpha =0.52$ in the lateral direction with a stiffness value of $4.6~\mathrm {pN\,nm^{-1}}$ which shows $\sim 84\%$, $\sim 120\%$, and $\sim 5\%$ improvement compared to the trapping of anatase, amorphous titania microspheres trapped by the RPB (Fig. 4j), and the titania core-shell with $\alpha =0.52$ trapped by the GB.

## Conclusion

In conclusion, it is shown that the core-shell microspheres have a great potential to enhance the optical trapping strength. With a proper core-shell thickness ratio, microparticles exhibit an anti-reflection property that dramatically reduces the scattering force and leads to stronger optical traps. Moreover, utilizing the structured light beams in place of the Gaussian beam to trap the core-shell particles result in rather stronger optical traps. We showed that the titania anti-reflection-coated microspheres provide stronger trap stiffness than the PS-silica particles in both axial and lateral directions. Additionally, using a proper core-shell ratio for both PS-silica and anatase-amorphous titania microspheres, the structured light beams provide stronger traps in the axial direction compared to the GB. For instance, for the PS-silica microsphere, the axial trapping stiffness of the PB is two times greater than that of the GB, while for the titania core-shell microspheres, the axial trapping stiffness of the RPB is $\sim 40\%$ higher than the corresponding value for the GB, at their optimal core-shell thickness ratios. Moreover, we showed that the RPB provides the strongest trap for titania core-shells in both the axial and lateral directions.
